# Generation of a novel Antibody-Drug Conjugate targeting endosialin: potent and durable antitumor response in sarcoma

**DOI:** 10.18632/oncotarget.19499

**Published:** 2017-07-22

**Authors:** Emily Capone, Enza Piccolo, Imma Fichera, Paolo Ciufici, Daniela Barcaroli, Arturo Sala, Vincenzo De Laurenzi, Valentina Iacobelli, Stefano Iacobelli, Gianluca Sala

**Affiliations:** ^1^ MediaPharma s.r.l., 66100, Chieti, Italy; ^2^ Dipartimento di Scienze Mediche, Orali e Biotecnologiche; University “G. d'Annunzio” Chieti-Pescara, Centro Studi sull'Invecchiamento, CESI-MeT, 66100 - Chieti, Italy; ^3^ Dipartimento di Scienze Psicologiche, della Salute e del Territorio, University “G. d'Annunzio” Chieti-Pescara, Centro Studi sull'Invecchiamento, CESI-MeT, 66100 - Chieti, Italy; ^4^ College of Health and Life Sciences, Brunel University London, UK; ^5^ Department of Gynecology and Obstetrics, University of Rome “La Sapienza”, 00100 - Rome, Italy; ^6^ Current address: Nouscom SRL 100 I-00128 Rome, Italy

**Keywords:** endosialin, ADC, sarcoma, duocarmycin, target therapy

## Abstract

The endosialin/CD248/TEM1 receptor is expressed on the cell surface of tumor-associated stroma cells as well as in sarcoma and neuroblastoma cells. This receptor is emerging as an attractive molecule in diagnostics and therapeutics because of its expression across the stroma of many human tumors, the low to absent expression in normal tissues and accessibility from the vascular circulation. In this study, we present evidence of the preclinical efficacy of a novel Antibody-Drug Conjugate (ENDOS/ADC). It consists of a humanized endosialin monoclonal antibody, named hMP-E-8.3, conjugated to a potent duocarmycin derivative. In endosialin expressing cancer cell lines, this ENDOS/ADC showed a powerful, specific and target-dependent killing activity. High expression levels of endosialin in cells correlated with efficient internalization and cytotoxic effects *in vitro*. Efficacy studies demonstrated that ENDOS/ADC treatment led to a long-lasting tumor growth inhibition of a cell line-based model of human osteosarcoma. Taken together, our results demonstrate that endosialin is an attractive target in sarcoma and suggest that ENDOS/ADC has the potential to be developed into a bio-therapeutic agent for these malignancies.

## INTRODUCTION

Endosialin (aka Tumor endothelial marker 1) is a cell surface protein encoded by the *CD248* gene in humans, belonging to a family of C-type lectin transmembrane receptors. Endosialin expression is essentially restricted to activated cells of the mesenchymal lineage, including pericytes and myofibroblasts during embryogenesis [1–3]. Importantly, expression of endosialin is dramatically reduced in adults, but has been shown to be up-regulated during pathologic states, including tumor progression and metastasis [4, 5] making endosialin an oncofetal protein with potential as therapeutic target [6, 7].

Several reports have documented that endosialin is frequently expressed in human cancers. However, the extent and the intensity of expression varies among the different cancer subtypes, as well as individual cells composing the neoplastic mass [8]. In a large series of 514 human sarcomas including undifferentiated pleomorphic sarcomas, rhabdomyosarcoma, synovial sarcomas, adult fibrosarcoma/spindle cell sarcoma and leiomyosarcomas, more than half of the tumours displayed endosialin on neoplastic sarcoma cells, whereas, in the remaining cases endosialin expression was restricted to pericytes and stromal fibroblasts [9]. Likewise, in epithelial neoplasms of other lineages, including colorectal, breast, histiocytomas, highly invasive glioblastoma, anaplastic astrocytomas, and metastatic melanomas, expression of endosialin was confined to pericytes and stromal fibroblasts [8, 10, 11].

Sarcomas are a heterogeneous group of malignant tumors that share similar embryologic derivation (predominantly mesodermal), pathologic appearance, and clinical presentation and behavior. Many sarcomas behave aggressively, and surgery with or without radiation remains the mainstay of management for localized disease, as most are resistant to chemotherapy. As many sarcomas occur in sites where adequate surgical clearance is not possible, and the prognosis is generally poor for recurrent and metastatic disease, the need for novel rationally selected therapies is urgent.

Antibody-Drug Conjugates (ADCs) share the common feature of targeting internalizing cell surface proteins with an antibody covalently linked to a highly potent cytotoxic compound [12–14]. In principle, this enables higher local exposure of the tumor to the drug than that permissible by systemic delivery of the free drug. Recent advances in this technology have resulted in some very encouraging objective clinical responses, and numerous ADCs are now in various stages of development [15, 16]. In this study, we generated and characterized monoclonal antibodies against the extra-cellular domain of human endosialin. One of the antibodies, hMP-E-8.3, was coupled via a peptidic linker to a potent duocarmycin derivative to generate an ADC. In endosialin expressing cancer cell lines, this novel ADC displayed specific and target-dependent killing activity and long-lasting tumor growth inhibition of cell line-based models of human sarcoma.

## RESULTS

Fourteen murine monoclonal antibodies, recognizing the ECD of human endosialin were generated as described in the method section. Antibodies were screened for *in vitro* binding and cell binding by ELISA or fluorescence-activated cell sorting (FACS) analysis. One murine antibody, mMP-E-8.3 was selected as lead compound displaying the highest affinity for the target protein (data not shown). The murine antibody was chimerized and after target binding validation, its CDRs were grafted into a human IgG1 isotype antibody framework (Supplementary Figure 1).

### hMP-E-8.3 antibody binding and internalization

**Figure 1:**
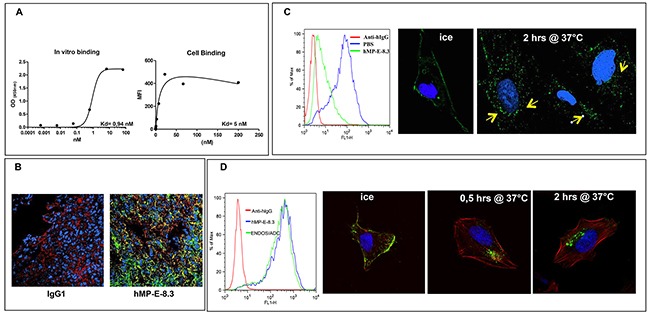
***hMP-E-8. 3 binds endosialin in vitro/in vivo and is internalized into SJSA-1 cells*** **(A)** Binding of hMP-E-8.3 to human recombinant endosialin's ECD by ELISA (*left*) or FACS analysis on living osteosarcoma SJSA-1 cells (*right*). **(B)** Binding of hMP-E-8.3 to endosialin *in vivo* as evaluated by immunofluorescence analysis of SJSA-1 tumor xenografts. Animals received a single injection of human IgG (as a control), or hMP-E-8.3, both at the dose of 10 mg/Kg. Twenty-four later, animals were sacrificed, the tumors frozen and tumor sections stained with AlexaFluor-488 conjugated anti-human IgG (*green*), a commercial antibody against human endosialin followed by AlexaFluor-546 conjugated anti-rabbit antibody staining *(red*) or Draq5 (*blue*). **(C)** Flow cytometry analysis (*left panel*) of endosialin surface expression in SJSA-1 cells cultured in the presence of hMP-E-8.3 (10 μg/ml) at 37°C for 2 hrs. The antibody is efficiently internalized as revealed by a marked reduction of endosialin expression (85% in 2 hrs). Fluorescence immunocytochemistry (*right panels*) of hMP-E-8.3 internalization in SJSA-1 cells. Cells were incubated with hMP-E-8.3 on ice for 20 min, then shifted at 37°C for 2 hrs. After harvesting, cells were stained with AlexaFluor-488 conjugated anti-human IgG (*green*). Draq5 (*blue*) was used to visualize nuclei. Yellow arrows indicate intracellular staining. Images were acquired with LSM-510 laser scanning confocal microscopy. **(D)** Flow cytometry analysis (*left panel*) of endosialin surface expression in SJSA-1 cells as evaluated by naked hMP-E-8.3 antibody or ENDOS/ADC. Confocal imaging (*right panels*) of ENDOS/ADC internalization in SJSA-1 cells. Cells were incubated with ENDOS/ADC (10 μg/ml) on ice for 20 min, then shifted at 37°C for the indicated times. After harvesting, cells were stained with AlexaFluor-488 conjugated anti-human IgG (*green*), Rhodamine-labeled phalloidin was used to visualize actin cytoskeleton (*red*), Draq 5 (*blue*) for nuclei staining.

The antibody bound human endosialin with affinity in the nanomolar range (K_D_ 0.94 to 5.0 nM), as determined using solid phase ELISA on human recombinant endosialin's ECD and FACS analysis on live SJSA-1 cells (Figure 1A). The antibody did not recognize murine endosialin; however it cross-reacted with cynomolgus's placenta where endosialin membrane staining is observed on decidual cells (Supplementary Figure 2A-2B).

To evaluate the ability of hMP-E-8.3 to bind endosialin *in vivo*, SJSA-1 cells were grown as xenograft tumors in nude mice. After the tumors became about 400 mm^3^ in volume, animals were injected with 250 μg of hMP-E-8.3. Twenty-four hours later, tumors were excised, snap frozen and sections stained with AlexaFluor-488 conjugated anti-human IgG. A substantial uptake of the antibody was observed in tumor tissues of animals injected with hMP-E-8.3 but not with a control isotype matched IgG (Figure 1B).

To determine whether hMP-E-8.3 internalized upon binding to the cell surface, and therefore represented a candidate for cytotoxic payload conjugation, SJSA-1 cells were exposed for 2 hrs to hMP-E-8.3 (10 μg /ml) or PBS and analyzed for endosialin expression by FACS. A marked (85%) reduction of surface expression of endosialin was observed in antibody treated cells (Figure 1C left panel). Antibody internalization was confirmed by confocal imaging, showing a clear de-localization of the hMP-E-8.3 staining from the cell surface to the perinuclear intracellular compartment upon shifting the cells from ice to 37°C (Figure 1C, right panels). These results indicate that hMP-E-8.3 quickly and efficiently internalized in endosialin expressing cells.

### Generation of ADC and *in vitro* cytotoxicity

A potent, soluble minor groove DNA binder (MGB) duocarmycin derivative bearing an enzymatic peptidic cleavable linker (valine-citrulline) was conjugated to monoclonal antibody hMP-E-8.3 by partial reducing the antibody and conjugating the drug to the available reduced inter-chain cysteine residues to produce ENDOS/ADC (Supplementary Figure 3A). The reaction conditions were optimized to produce a monomeric ADC with a DAR of 3.5-4.0 (Supplementary Figure 3B-3C).

As first step, we compared the binding affinity of the ENDOS/ADC and naked hMP-E-8.3 antibody. SJSA-1 human osteosarcoma cells were chosen, as these cells expressed the highest levels of endosialin. FACS analysis performed on live SJSA-1 cells revealed no difference in binding between naked antibody and ENDOS/ADC (Figure 1D, left panel) indicating that the conjugation process did not alter the ability of the antibody to bind his target on cell surface. ENDOS/ADC internalization ability in SJSA-1 cells was confirmed by confocal imaging. Of note, a marked accumulation of ENDOS/ADC occurred already 0.5 hr after shifting cells from ice to 37°C (Figure 1D, right panels).

**Figure 2:**
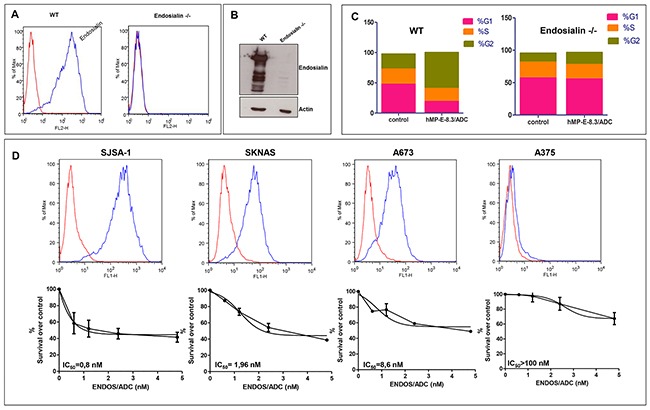
ENDOS/ADC *in vitro* cytotoxic activity correlates with endosialin expression Endosialin −/− SJSA-1 cells were generated by CRISPR-Cas9 system of genome editing. Lack of endosialin expression was documented by **(A)** FACS analysis and **(B)** Western blotting. **(C)** Cell cycle analysis was performed after exposure of wild-type or endosialin −/− SJSA-1 cells to 0.4 μg/ml of ENDOS/ADC. **(D)** Levels of endosialin surface expression determined by cytometry analysis using hMP-E-8.3 antibody for cells staining (upper panel). ENDOS/ADC cell killing activity (lower panel) after 120hrs of cells exposure to increasing doses of ENDOS/ADC. The following human cancer cell lines and tissue of origin were used: SJSA-1, osteosarcoma; SKNAS, neuroblastoma; A673 Ewing's sarcoma; A375 melanoma. The values are expressed as mean ± SD of three experiments. IC50 and best-fit curve were calculated using GraphPad Prism 5 software.

As a proof-of-concept of ENDOS/ADC activity *in vitro*, cell cycle analysis of ADC-treated SJSA-1 cells was evaluated and compared to cells treated with PBS. Additionally, as a control of specificity, SJSA-1 endosialin −/− cells were generated by CRISPR/CAS 9 technology and assayed for cytotoxicity upon exposure to the ADC. The induced mutation in SJSA-1 endosialin −/− cells was validated by genomic DNA sequencing and the absence of expression of endosialin was confirmed by FACS analysis and western blotting (Figure 2A–2B). As expected, a complete S-G2 block was observed in wild-type cells after 48 hrs exposure to ENDOS/ADC; the block was completely absent in endosialin −/− cells (Figure 2C). These data clearly indicated that hMP-E-8.3 antibody was able to deliver and release the conjugated payload into cancer cells in a target-dependent manner. Next, we tested the cytotoxic activity of ENDOS/ADC in four human tumor cell lines expressing different levels of endosialin: high (osteosarcoma SJSA-1), intermediate (neuroblastoma SKNAS and Ewing's sarcoma A-673), or not expressing endosialin (metastatic A375 melanoma cells) (Figure 2D upper panel). SJSA-1 cells, which display the higher endosialin expression level, were the most sensitive to ENDOS/ADC with an IC_50_ = 0.8 nM; SKNAS and A-673 were also sensitive, with IC_50_ of 1.96 and 8.6 nM, respectively, whereas A375 were resistant, as expected (IC_50_ > 100 nM) (Figure 2D lower panel).

These results indicate that the growth inhibitory activity of ENDOS/ADC correlates with the amount of target expression on the cell surface.

### ADC efficacy and safety in an *in vivo* human osteosarcoma model

**Figure 3:**
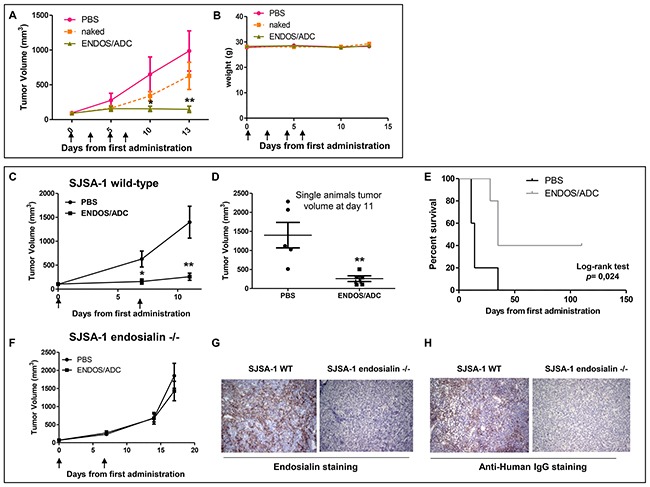
Therapeutic activity of ENDOS/ADC against SJSA-1 osteosarcoma xenografts **(A)** Mice were implanted subcutaneously with 2×10^6^ SJSA-1 cells. When tumors reached a volume of ∼100 mm^3^, mice were randomly grouped (N=5) and intravenously injected every 3 days with vehicle (PBS), naked hMP-E-8.3 antibody (10 mg/kg), or ENDOS/ADC (10 mg/kg) for a total of 4 injections. **(B)** No significant treatment-related body weight loss was registered. **(C)** Growth curves of SJSA-1 tumor xenografts treated with a reduced schedule (two injections of 10 mg/kg ENDOS/ADC one week a part). **(D)** Plot of single animals tumor volumes at day 11th. **(E)** Survival curve evaluated by Kaplan-Meier and analyzed by the log-rank test. **(F)** Growth curves of endosialin −/− SJSA-1 derived xenografts treated as in C. IHC staining of endosialin **(G)** or ENDOS/ADC **(H)** in wild-type or endosialin −/− SJSA-1 derived xenografts. * *p* < 0.05, ** *p* < 0.01.

The therapeutic efficacy of ENDOS/ADC was evaluated in a human osteosarcoma SJSA-1 derived xenograft model. When tumors reached an average volume of around 100mm^3^, animals were divided in three arms and treated with a total of four injections (once every three days) with naked hMP-E-8.3 antibody (10 mg/kg), ENDOS/ADC (10 mg/kg), or PBS (as a control group). The *naked* antibody slightly reduced tumor growth, although reduction was not statistically significant, whilst a significant tumor remission was observed in mice treated with ENDOS/ADC (Figure 3A). No differences in terms of body weight loss or tumor tissues cellular architecture was observed in any of the treatment groups (Figure 3B and Supplementary Figure 3D). A reduced treatment schedule, i.e a total of two injections a week apart was similarly effective in producing a complete tumor remission (Figure 3C). Of note, at day 11^th^ two out of five mice showed a complete remission, resulting in a prolonged survival of 100+ days after the start of the treatment. (Log-rank test p= 0.02, Figure 3D-3E). On the contrary, no tumor response was observed in endosialin −/− derived xenografts (Figure 3F). The lack of expression of endosialin in xenografts derived from endosialin −/− cells and the accumulation of ENDOS/ADC in wild-type cells were confirmed by immunohistochemistry (IHC) (Figure 3G–3H). These results show that the therapeutic activity of ENDOS/ADC is specific and dependent on the target.

## DISCUSSION

Osteosarcomas and more in general sarcomas are malignant tumors that most commonly affect children, adolescents, and young adults. Despite advances in the treatment with the introduction of new surgical techniques and innovative drugs, these tumors can be hard to treat and novel therapies are urgently needed.

ADCs are an emerging new class of targeted therapeutics that are particularly attractive because they combine the selectivity of antibodies toward the target with the potent cell killing activity of cytotoxic drugs [13, 17]. ADCs can deliver these drugs in the picomolar range directly inside the tumor cells. To date, there are two FDA approved ADCs and more than thirty in clinical trials [14]. In order to ensure the required specificity, it is necessary that the armed antibody is directed to a target which is abundant on the surface of cancer cells, but not normal cells.

Endosialin is a cell surface receptor detected in mesenchymal tumors, such as sarcoma, neuroblastoma other than in perivascular and tumor-associated stroma. The exact role of endosialin is still largely unknown. However, its implication in tumour neoangiogenesis and the observed high expression levels in malignant cells has stimulated the development of therapeutics targeting this receptor. Importantly, normal adult tissues do not express endosialin, making it an ideal target for an ADC based therapy.

In this study, we have generated an ADC targeting endosialin based on a novel humanized anti-endosialin antibody, hMP-E-8.3. Compared to the naked antibody, ENDOS/ADC displayed similar binding affinity for the target. The novel ADC was endowed with a marked and durable antitumor activity which is dependent on target expression. Other antibodies targeting endosialin have been developed, mainly with the aim of targeting endosialin expression in the vasculature and stromal cells of carcinomas [18–21]. Recently, an anti-endosialin/Monomethyl Auristatin E (MMAE) conjugate has been generated and found to be effective in targeting neuroblastoma and Ewing sarcoma in preclinical *in vitro* and *in vivo* models [22]. In our study, ENDOS/ADC treatment produced a significant tumor response with only two injections, one week apart, in a model of osteosarcoma (SJSA-1). Besides the different tumor models used, the key distinction between these two reports lies in the different nature of the payload, a DNA alkylating agent *versus* a microtubule disruptor.

The present proof-of-concept study demonstrates that endosialin positive osteosarcomas can be specifically and effectively targeted by the anti-endosialin antibody hMP-E-8.3 conjugated to a potent MGB duocarmycin derivative. The therapeutic efficacy demonstrated by this ADC deserves further preclinical testing in endosialin positive malignancies other than osteosarcomas.

## MATERIALS AND METHODS

### Reagents

As positive control for mouse cross reactivity assays was used the commercial rabbit anti-endosialin antibody 18160-1-AP from Proteintech (Proteintech Group Inc., Chicago, IL, USA) which has been reported to bind murine endosialin [23].

Actin antibody from Sigma-Aldrich (St Louis, MO, USA). 3-(4,5-Dimethylthiazole-2-yl)-2,5-diphenyl-tetrazolium bromide (MTT) was purchased from Sigma (Sigma Aldrich Corporation, St. Louis, MO, USA). Recombinant human endosialin (CD248) was purchased from R&D (R&D Systems, Inc., Minneapolis, MN, USA).

### Cell lines

Sarcoma (SJSA-1), melanoma (A375m), neuroblastoma (SKNAS) and Ewing sarcoma (A673) cells were purchased from American Type Culture Collection (Rockville, MD, USA). The cells were cultured according to manufacturer's instructions, in a medium supplemented with 10% heat-inactivated fetal bovine serum (FBS; Invitrogen), l-glutamine, 100 units/ml penicillin, and 100 μg/ml streptomycin (Sigma-Aldrich Corporation, St. Louis, MO, USA), and incubated at 37°C in humidified air with 5% CO_2_.

### Generation and characterization of anti-endosialin antibodies

Four-weeks old Balb/c mice were immunized by intraperitoneal injection as emulsions in Complete Freund's Adjuvant (CFA) or Incomplete Freund's Adjuvant (IFA). Seven days later, mice were given an additional intraperitoneal injection of the immunogen consisting of a mixture of peptides with specificity for the extra-cellular domain of endosialin, as reported in Supplemental Figure 1 (sequence of peptide of mMP-E-8.3 highlighted). After additional seven days, mice were boosted intravenously with the immunogen, and spleens were removed for cell fusion 3 days later. Somatic cell hybrids were prepared by fusion of immune splenocytes with the murine nonsecreting myeloma cell line NS-1 by standard procedures [24]. Hybridoma cell lines expressing antibodies recognizing the ECD of human recombinant endosialin in ELISA were subcloned four to six times to ensure that the cell line was monoclonal and stable. Monoclonal hybridoma cell lines were grown in RPMI 1640 medium supplemented with 5% fetal calf serum (Invitrogen, Carlsbad, CA, USA) for 2–3 weeks. IgG were purified over protein G-Sepharose 4 Fast Flow (Amersham Biosciences, Little Chalfont, UK) according to standard procedures [25].

Binding and affinity of the antibodies was determined by solid phase ELISA using immobilized human ECD recombinant and FACS analysis using live SJSA-1 human osteosarcoma cells, respectively. The concentration of antibody required for obtain 50% of maximal binding was considered the Kd value. The antibody displaying the highest affinity was selected as lead compound and named mMP-E-8.3.

### Antibody humanization

The mMP-E-8.3 antibody was humanized by CDRs grafting into an IgG1 scaffold as previously described [26]. Briefly, in a first step a chimeric variant of the mMP-E-8.3 antibody was generated by fusing the VH (variable domain of the heavy chain) and VL (variable domain of the light chain) of the murine antibody to the corresponding human constant domains. Successively, humanized MP-E-8.3 variants were generated by identifying murine complementary determinant regions (CDR) which were grafted onto a human antibody framework. Recombinant genes were placed into the pCDNA3.1 expression vector and transfected into Chinese Hamster Ovary-S (CHO) cells. Antibody variants were screened for antigen binding affinity by ELISA and FACS and lead candidate was selected and named hMP-E-8.3.

For small/medium scale production of antibody variants, transiently transfected CHO cells were grown using a benchtop BioFlo 3000 bioreactor (New Brunswick Scientific); antibody-containing supernatants were purified by ultrafiltration (VivaFlow-200 membrane Sartorium stedim Biotech) and immune selection on a Protein-A (Pall Protein A ceramic HyperD F) affinity matrix. The selected hMP-E-8.3 antibody is a G1m17 IgG1 allotype with a human km3 kappa LC.

### ELISA

Human recombinant endosialin (1 μg/ml) was pre-coated overnight at 4°C on 96 well-plates NUNC Maxisorp modules. After blocking with 1% BSA in PBS containing 0,05 % Tween-20 for 1 hour at room temperature, mMP-E-8.3 or hMP-E-8.3 was added and incubated at the indicated concentration for 2 hours at room temperature. After several washes with PBS-0,05 % Tween-20, anti-mouse IgG-HRP or anti-human IgG-HRP (from Bio-Rad; Hercules, California, USA) were added and incubated for 1 hour at room temperature. After washes, stabilized chromogen was added for at least 10 minutes in the dark, before stopping the reaction with the addition of 1 N H_2_SO_4_. The resulting color was read at 450 nm with an Elisa reader.

### FACS analysis

Endosialin cell surface expression was analyzed by flow cytometry using a FACSCalibur cytometer (Becton Dickinson, Buccinasco, MI, Italy). Briefly, around one million of proliferating cells were harvested and labeled with hMP-E-8.3 on ice for 20 minutes. Cells were then washed with 2 ml PBS, pulled down, and stained with an Alexa Fluor 488 Goat anti-Human antibody (Molecular Probes, Life Technologies, Paisley, UK). The data were analyzed using CELLQuest 3.2.1.f1 software (Becton Dickinson).

Cell Cycle was analyzed by flow cytometric evaluation of DNA content according to the Nicoletti method [27]. 2×10^6^ cells were harvested by centrifugation at 800g for 5 min and fixed in 70% ethanol at 4°C overnight. Cells were then washed twice with PBS and stained in 0.5 ml PBS containing Propidium iodide (PI, 50 μg/ml) and RNAase (10 μg/ml) 4°C overnight in the dark. 20,000 singlet gated events were acquired using a FACSCanto II cytometer (Becton Dickinson, Buccinasco, MI, Italy). Finally, data were analyzed using FLOW JO, LLC software.

### ADC preparation

A potent, soluble Minor Groove DNA Binder (MGB) duocarmycin derivative bearing an enzymatic peptidic cleavable linker (valine-citrulline) was conjugated to hMP-E-8.3 by partial reducing the antibody and conjugating the drug to the available reduced interchain cysteine residues.

The product was characterized by SDS-PAGE under reducing and not reducing conditions, size exclusion chromatography (SEC) to determine the aggregate state, hydrophobic interaction chromatography (HIC) to evaluate the presence of different loaded isoforms in native conditions, and PRLP LC/MS in reducing conditions to determine the Drug to Antibody Ratio (DAR). The sample was totally reduced to separate Light and Heavy chains before submitting to LC/MS analysis. The MW of the single species was determined by mass spectrometry on a single quadrupole instrument Agilent 1946. The presence of the drug was confirmed by absorbance at 320 nm in the chromatographic peaks and DAR calculated by the ratio between 320nm/280nm absorbancies.

### Endosialin gene disruption by CRISPR-Cas9 system

Endosialin expression was ablated in SJSA-1 cells by means of CRISPR-Cas9 system of genome editing in accordance with the protocol developed by Zhang and co-workers [28, 29].

#### Target selection for sgRNA

A 20 nucleotide sgRNA (single guide RNA) target sequence (AAGGGCGTGGTATACACGGC), immediately followed by a PAM sequence (TGG) for S. piogenes Cas-9, was identified into the antisense strand of endosialin locus, through the CRISPR design tool (crispr.mit.edu).

#### Generation of single guide expression plasmid

Endosialin CRISPR plasmid was generated by cloning annealed oligos for target sequence into the BbsI restriction site of pX330 (Addgene 42230), bearing both sgRNA scaffold and S. piogenes Cas-9 endonuclease.

#### Cells transfection and isolation of clonal cell lines by FACS

Endosialin CRISPR was transiently co-tranfected with a GFP expression plasmid in SJSA-1 cells using the Calcium Phosphate transfection Kit, (ThermoFisher Scientific; Waltham, Massachussets, USA). 48 hrs after transfection, single GFP-positive cells were isolated by cell sorting (FACS ARIA III, BD Biosciences, San Jose, CA) and seeded into 96 well plates.

#### Identification of endosialin KO cell line

Colonies arising from a single cell were replica plated and genomic DNA was harvested using QuickExtract (EpiCentre). The target region was PCR amplified (Fw primer: 5′-tacctgtgccagtttggcttc-3′; Rev 5′- tgcttcacgcagagcagagag -3′) and amplification bands were cloned using the TOPO® TA kit [Invitrogen], Sanger sequenced (Eurofins genomics) and aligned to observe the individual mutations and identify KO clones. Sequencing shows two base-pair deletion in one allele and twenty-five base-pair deletion in the other, both resulting in a frame-shift. All oligonucleotides were synthesized by Integrated DNA Technologies (IDT).

### Internalization assays

SJSA-1 cells were plated in 12 well-plates and grown in 10% FBS RPMI-1640 for 24 hours. Cells were then incubated with 10 μg/ml of hMP-E-8.3 for 30 minutes on ice and returned at 37°C for 2 hours. After 2 hours, cells were stained with a goat anti-human Alexa 488-conjugated secondary antibody (ThermoFisher Scientific; Waltham, Massachussets, USA) and samples were analyzed by flow cytometry using a FACSCalibur cytometer (Becton Dickinson, Buccinasco, MI, Italy). Finally, data were analyzed using CELLQuest 3.2.1.f1 software (Becton Dickinson). For confocal imaging, after 0.5 or 2 hours, cells were fixed in 4% paraformaldehyde for 15 minutes at room temperature, permeabilized with 0.25% Triton-X100 in PBS for 5 minutes, and blocked with 5% goat serum in PBS for 30 minutes at room temperature. Then cells were stained for 1 hour at room temperature with a goat anti-human Alexa 488-conjugated secondary antibody (Molecular Probes, Life Technologies, Paisley, UK) (green staining) and with Rhodamine-labelled phalloidin (Sigma-Aldrich Corporation, St. Louis, MO, USA). Cell nuclei were counterstained in blue using DRAQ5 (Cell Signaling Technology, Danvers, MA, USA). Images were acquired with a Zeiss LSM 510 meta-confocal microscope (Zeiss, Oberkochen, Germany) using 488-, 543-, and 633-nm lasers.

### Cell viability assay

Cells were seeded at a density of 7 × 10^4^ cells/well in 24-well plates in 10% FBS containing medium and allowed to attach and propagate overnight before the treatments. The cells were washed once with pre-warmed phosphate-buffered saline (PBS) and exposed to the specified experimental conditions as indicated. After treatment, cells were incubated with MTT (final concentration of 0.5 mg/ml) at 37°C for 2 hours. Spectrophotometric absorbance of the samples was determined at 570 nm. All experiments were performed in quadruplicate.

### *In vivo* tumor growth

Athymic CD-1 nu/nu mice (5-7 weeks old) were purchased from Charles River Laboratories (Calco, LC, Italy) and maintained under specific pathogen-free conditions with food and water provided *ad libitum* and the animals' health status was monitored daily. Procedures involving animal and their care were established according to the institutional guidelines in compliance with national and international policies (Authorization n° 973/2016-PR).

For *in vivo* ADC testing, human osteosarcoma cancer xenografts were established by implanting subcutaneously 2×10^6^ SJSA-1 cells in 5-week old CD1 female nude mice. When xenografts became palpable (with a tumor volume average of about 100 mm^3^), mice were divided in a manner to provide a similar range of tumor size in each group. Tumor volumes were monitored by a caliper every week and volumes calculated according to the following formula: tumor volume = (length * width^2^)/2. Mean volumes of treated and untreated xenografts were compared using an unpaired t test (Student's t test) considering as statistically significant a P value < 0.05 (CI 95%). A tumor volume of 2 cm^3^ was chosen as endpoint for both experiments after which mice were sacrificed. Survival curves were derived from Kaplan-Meier estimates and compared by log-rank test (GraphPad Prism 5).

### Immunohistochemistry

For endosialin staining of murine stomach and tumor tissues from xenografts experiments, tissues were fixed in 4% buffered formalin for 24 hours at room temperature. The fixed tissues were then dehydrated in ethanol, cleared in bioclear and then embedded in paraffin. Sections were cut to a thickness of 3 μm and taken on positively charged slides. Tissue sections were rehydrated in a graded alcohol series and rinsed in running tap water. Epitope retrieval was achieved by boiling the tissue sections in sodium citrate buffer (0.01 M, pH 6), in a microwave owen for 10 minutes. Slides were rinsed in distilled water and then in Tris-buffered saline (0.05 M, pH 7.6).

Slides were preincubated with 1% bovine serum albumin in Phosphate Buffer Solution (BSA/PBS) for 30 min at room temperature and then incubated with hMP-E-8.3 (5 μg/ml) overnight at 4°C. As a positive control, slides were incubated with a rabbit monoclonal antibody recognizing murine endosialin (Proteintech, 1:100 dilution). After washing, goat anti-human and goat anti rabbit, both HRP-conjugated were applied for 1 h at room temperature. Finally, slides were incubated with diaminobenzidine (DAB, DAKO) and coverslipped with an antifade-containing mounting medium (DAKO). To evaluate hMP-E-8.3 cross reactivity with monkey, a frozen tissue micro array (TMA) from US Biomax (# FCY 301a) was used and processed according to the manufacturer's protocol.

## SUPPLEMENTARY FIGURES



## Abbreviations

ADC: antibody-drug conjugate
MGB: Minor Groove DNA Binder
DAR: drug to antibody ratio
FACS: fluorescence-activated cell sorting
IHC: immunochemistry
TMA: tissue micro array
ECD: extra cellular domain
ELISA: enzyme-linked immunosorbent assay
