# Supplementary material for: Generation of a novel Antibody-Drug Conjugate targeting endosialin: potent and durable antitumor response in sarcoma
Source: Oncotarget. 2017 Jul 22;8(36):60368–77. doi: 10.18632/oncotarget.19499 (PMC5601145; doi:10.18632/oncotarget.19499)
Supplement: Supplementary file 1 [file oncotarget-08-60368-s001.pdf]

Generation of a novel Antibody-Drug Conjugate targeting endosialin: potent and durable antitumor response in sarcoma

SUPPLEMENTARY MATERIALS

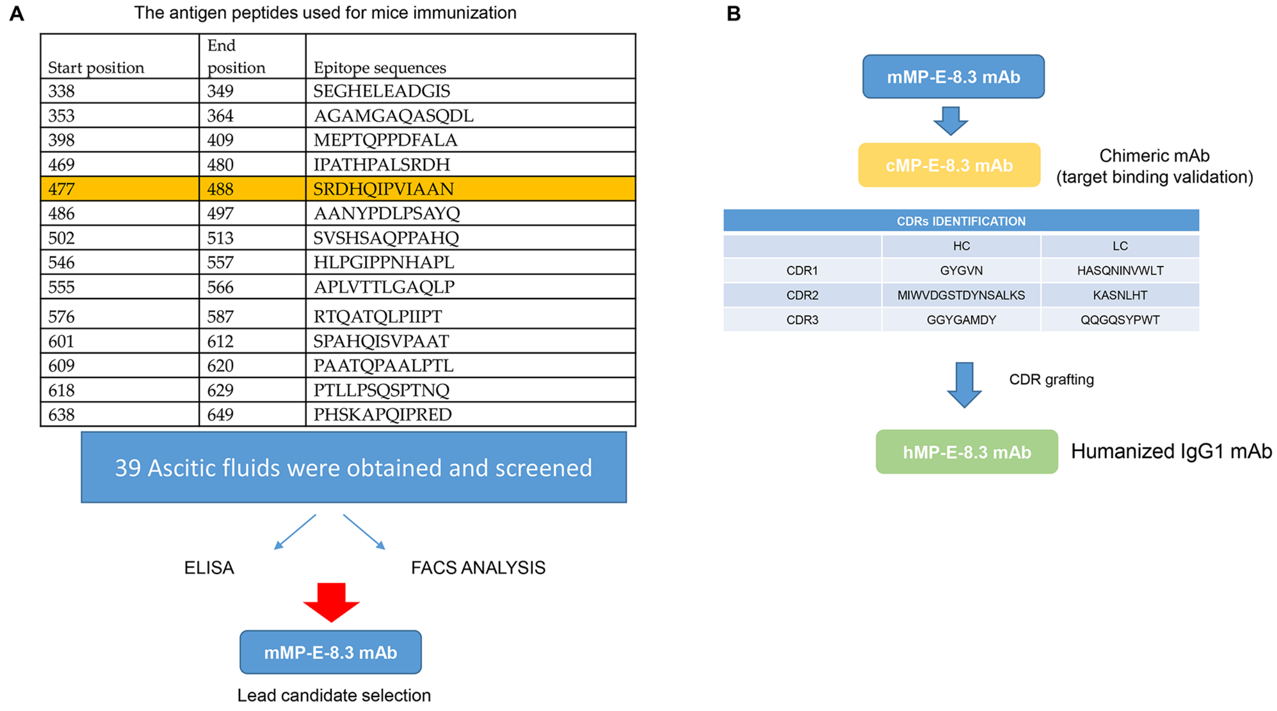

Start position	End position	Epitope sequences
338	349	SEGHELEADGIS
353	364	AGAMGAQASQDL
398	409	MEPTQPPDFALA
469	480	IPATHPALSRDH
477	488	SRDHQIPVIAAN
486	497	AANYPDLPSAYQ
502	513	SVSHSAQPPAHQ
546	557	HLPGIPPNHAPL
555	566	APLVTTLGAQLP
576	587	RTQATQLPIIPT
601	612	SPAHQISVPAAT
609	620	PAATQPAALPTL
618	629	PTLLPSQSPTNQ
638	649	PHSKAPQIPRED

CDRs IDENTIFICATION		
	HC	LC
CDR1	GYGVN	HASQNINVWLT
CDR2	MIWVDGSTDYNSALKS	KASNLHT
CDR3	GGYGAMDY	QQGQSYPWT

Supplementary Figure 1: Generation of hMP-E-8.3 antibody. (A) Balb/c mice were immunized by intraperitoneal injection of the immunogen consisting of a mixture of peptides with specificity for the ECD of endosialin (sequence of peptide of mMP-E-8.3 highlighted). The antibody displaying the highest affinity was selected as the lead and named mMP-E-8.3. **(B)** In a first step, a chimeric variant of the mMP-E-8.3 antibody was generated by fusing the VH and VL of the murine antibody to the corresponding human constant domains. After the validation of target binding, humanized MP-E-8.3 variants were generated by identifying murine complementary determinant regions (CDRs shown in the Table) which were grafted onto a human antibody framework. Humanized antibody variants were screened for antigen binding affinity by ELISA and FACS analysis and the lead candidate (based on the affinity for the target and the yield) selected and named hMP-E-8.3.

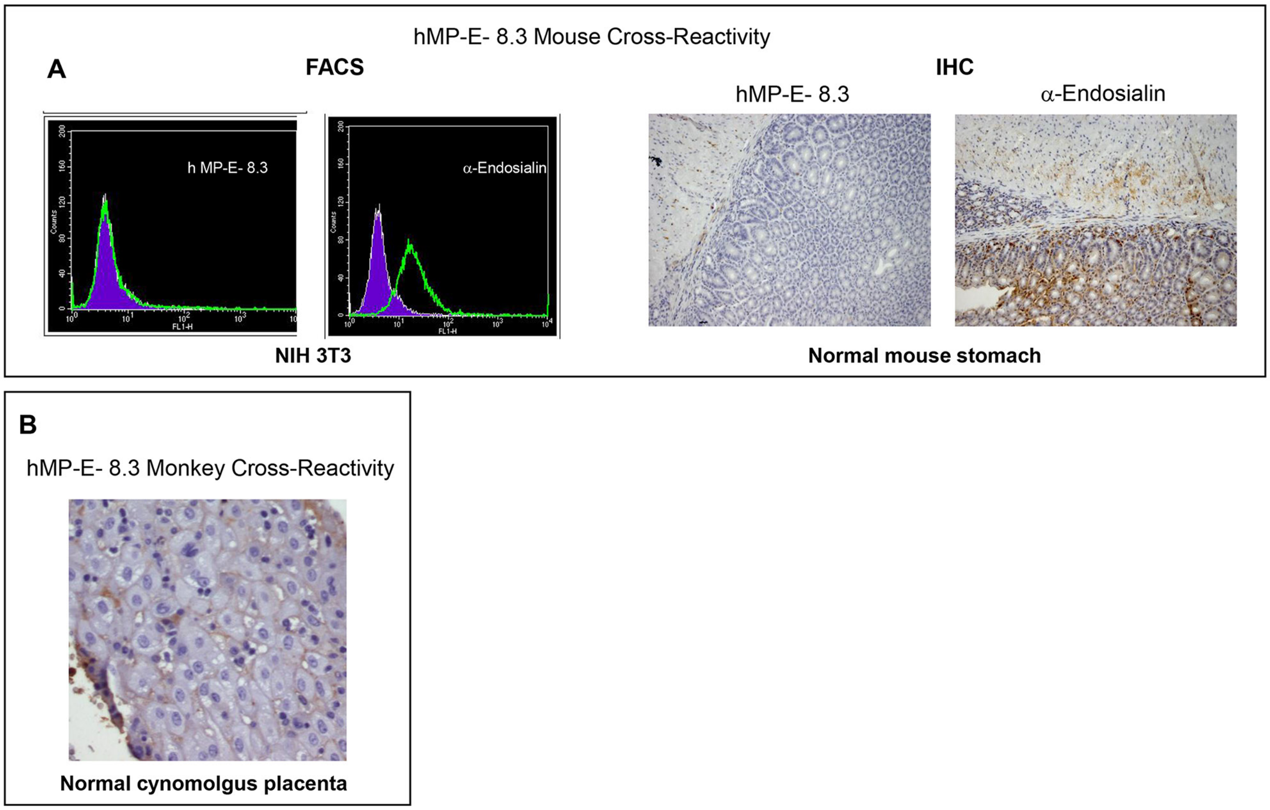

Supplementary Figure 2: hMP-E-8.3 mouse and monkey cross reactivity. (A) hMP-E-8.3 mouse cross reactivity was evaluated by FACS analysis (left) and IHC (right) using murine NIH3T3 fibroblast cells and sections of murine stomach tissue, respectively. **(B)** hMP-E-8.3 reactivity with cynomolgus monkey placenta tissues evaluated by IHC.

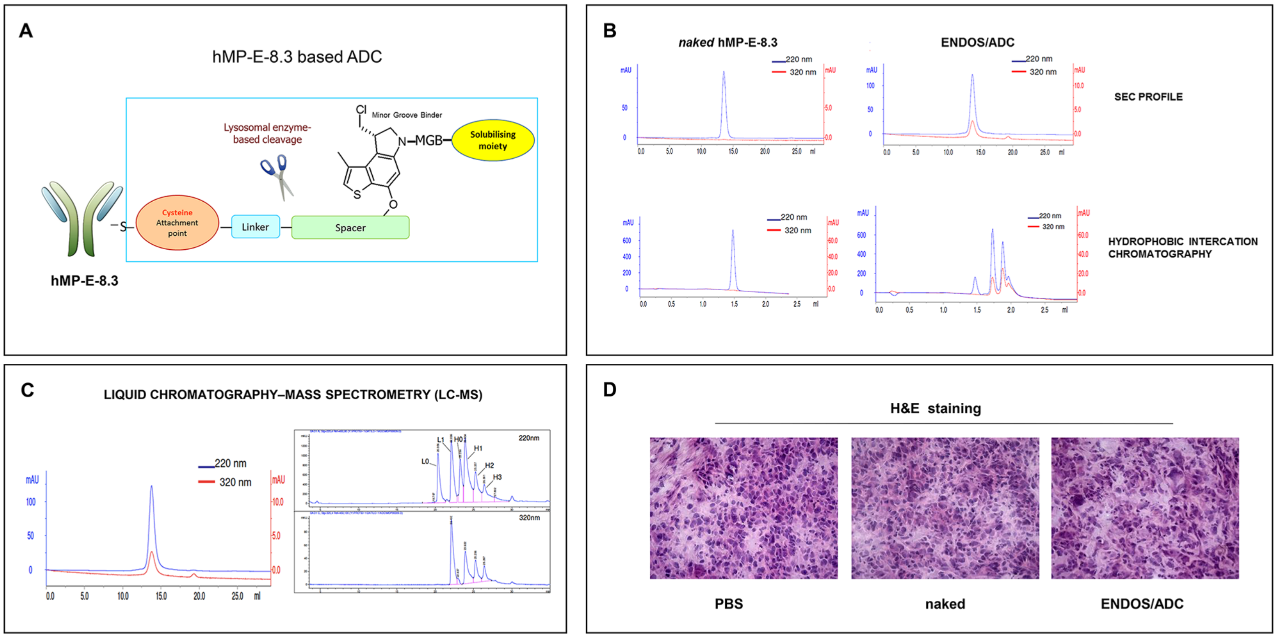

Supplementary Figure 3: ENDOS/ADC generation and characterization. **(A)** Schematic representation of ENDOS/ADC generation. A potent, soluble MGB duocarmycin derivative bearing a peptidic cleavable linker (Valine-Citrulline) was conjugated to hMP-E-8.3 antibody by sulfhydryl groups after partial interchain cysteine residues reduction. **(B)** Size exclusion chromatography (SEC) and hydrophobic interaction chromatography (HIC) to determine the aggregation state and the presence of different loaded isoforms in native conditions, respectively. **(C)** PRLP LC/MS in reducing conditions to determine the Antibody Drug Ratio (DAR). The presence of the drug was confirmed by absorbance at 320 nm in the chromatographic peaks and DAR calculated by the ratio between 320nm/280nm absorbances. In this specific example, the DAR was 3.7. **(D)** Hematoxylin and Eosin staining of tumor tissues from control and treated animals at the end of experiment.
